# Supplementary material for: Causal Relationship between Aging and Anorexia Nervosa: A White-Matter-Microstructure-Mediated Mendelian Randomization Analysis
Source: Biomedicines. 2024 Aug 16;12(8):1874. doi: 10.3390/biomedicines12081874 (PMC11351342; doi:10.3390/biomedicines12081874)
Supplement: Supplementary file 1 [file biomedicines-12-01874-s001.zip › Supplementary Figure/Figure S8/Leaveoneout plot of MR analysis for TL to ubm-b-2042.pdf]

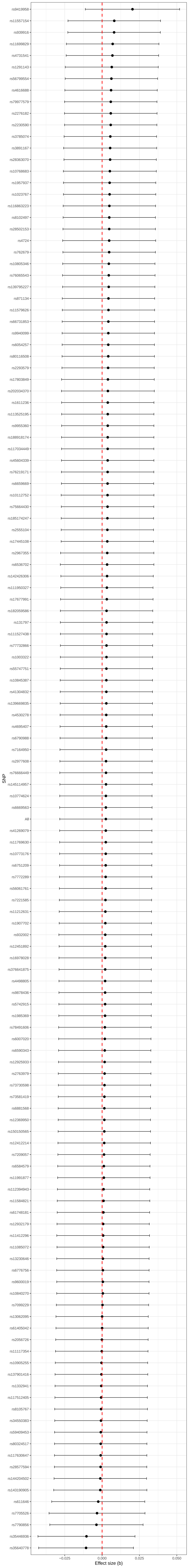
SNP
Effect size (b)
-0.025
0.000
0.025
0.050
rs9419958
rs11557154
rs939916
rs11699829
rs4731541
rs1291143
rs56799554
rs4616688
rs79977579
rs2276182
rs2230590
rs3785074
rs3891167
rs28363070
rs10768683
rs1957937
rs1023767
rs116863223
rs8102497
rs28502153
rs4724
rs762679
rs10805346
rs76065543
rs139795227
rs871134
rs11579626
rs66731853
rs9940099
rs6054257
rs80116508
rs2293579
rs17803849
rs202034370
rs1611236
rs113525195
rs9955360
rs188918174
rs117034449
rs45604339
rs76219171
rs6659669
rs10112752
rs75664430
rs185174247
rs2555104
rs17445108
rs2967355
rs6536702
rs142426306
rs111950327
rs17677991
rs182059586
rs131797
rs111527438
rs77732866
rs1003322
rs55747751
rs10845387
rs41304832
rs139669835
rs4530278
rs4695407
rs6790988
rs7164950
rs2977608
rs76666449
rs145114957
rs10774624
rs6669563
All
rs41269079
rs11769630
rs10773176
rs6751209
rs7772289
rs56061761
rs7221585
rs11212631
rs1907702
rs932002
rs12451892
rs16978028
rs376641875
rs4498805
rs9878436
rs5742915
rs1985369
rs78491606
rs6007020
rs6590343
rs12925933
rs2763979
rs73730598
rs73581419
rs6881568
rs12369950
rs150150565
rs12412214
rs7209057
rs6584579
rs11991877
rs112394943
rs11584821
rs61748181
rs12932179
rs11412296
rs11085072
rs13230646
rs6776756
rs9600019
rs10840270
rs7099229
rs13062095
rs61405042
rs2056726
rs11117354
rs10905255
rs137901416
rs1332941
rs117512405
rs8105767
rs34550383
rs59409453
rs80324517
rs117630647
rs28577594
rs144204502
rs143190905
rs611646
rs7705526
rs7790856
rs35446936
rs35640778
